# People with High Perceived Infectability Are More Likely to Spread Rumors in the Context of COVID-19: A Behavioral Immune System Perspective

**DOI:** 10.3390/ijerph20010703

**Published:** 2022-12-30

**Authors:** Qian Ding, Xingyu Luo

**Affiliations:** College of Education Science, Xinyang Normal University, Xinyang 464000, China

**Keywords:** COVID-19, perceived infectability, rumor trust, rumor spreading

## Abstract

Since the outbreak of COVID-19, many studies have explored the influencing factors of rumor spreading, such as anxiety, risk perception and information source credibility, but few studies have focused on the impact of individual differences. Based on the theory of behavioral immune systems, we investigated the impact of perceived infectability on rumor spreading and the mediating role of rumor trust in the context of COVID-19. Two studies were investigated using the scale and recall–report task of rumor spreading. The results show that perceived infectability was a significant positive predictor of rumor spreading. However, the impact of perceived infectability on rumor spreading was not direct, and it mainly indirectly affected rumor spreading through the mediating role of rumor trust. Overall, the findings suggest that individuals with high perceived infectability are more likely to believe rumors and then spread rumors during the epidemic. This study advances the literature on rumor spreading and behavioral immune systems and provides practical implications to anti-rumor campaigns.

## 1. Introduction

### 1.1. Background

Public health emergencies are a common challenge to mankind. The COVID-19 epidemic that swept the world has not only seriously threatened people’s health and brought significant economic recession but has also become a breeding ground for rumors to spread [1,2]. Rumors refer to unsubstantiated but widely circulated information in ambiguous, dangerous or potentially threatening circumstances [3]. With the development of social media, rumors can spread faster and wider than ever before [4]. The massive spread of rumors on social media not only destroys the news ecosystem but can also hinder people’s scientific protection behavior and aggravate their panic [5,6]. Therefore, it is very important to explore the factors impacting rumor spreading in the context of the epidemic.

Previous research has found that cognitive and emotional factors are the main objects of study for the influencing factors of epidemic rumors. For example, Sun et al. found that anxiety and perceptions of the serious consequences of spreading rumors could predict willingness to re-spread rumors [7]. Luo et al. thought that fear is an important factor affecting rumor spreading [8]. Hu et al. and Wang et al. believed that individuals with higher risk perception and negative emotions are more likely to spread rumors [9,10]. In fact, individual cognitive and emotional arousal are often closely related to individual traits. Nevertheless, few studies have explored the effect of individual differences on rumor spreading in public health emergencies.

The behavioral immune system (BIS) is a protective mechanism that has evolved in humans to reduce the risk of self-infection with a disease. It motivates individuals to take steps to avoid disease through a series of psychological processes, including cognitive and emotional processes, when a potential disease threat arises [11]. Because of the invisibility of pathogens, to maximize the recognition and avoidance of pathogen threats, the behavioral immune system has two characteristics: overgeneralization and functional flexibility. Overgeneralization, also known as the smoke detector principle, refers to the tendency of the behavioral immune system to overgeneralize or be overly sensitive to disease-related signals (e.g., elderly, obese and disabled) [12,13,14]. This is because the cost of misjudging a disease threat and engaging in avoidance is much less than the cost of ignoring a disease threat that leads to infection, which would directly threaten the need for individual safety [11,15].

Functional flexibility refers to whether the activation of the behavioral immune system is regulated by environmental cues and an individual’s susceptibility to pathogens. Avoiding a potential disease threat has both benefits (e.g., successful avoidance of infection) and costs (e.g., inability to meet the need for social interaction), and to maximize benefits, the behavioral immune system requires a degree of flexibility, as its activation is influenced by both environmental factors (contextual activation) and perceived disease threats (trait activation) [16].

This study focuses on the role of trait activation on rumor spreading. There are trait-level differences in individuals’ propensity to avoid disease due to genetic, developmental and childhood illness histories [15,17]. For example, individuals who are frequently ill perceive themselves to be more susceptible to disease and therefore have lower thresholds of activation of their behavioral immune systems than those of individuals without a history of illness. Trait activation of the behavioral immune system may have an independent effect under the general influence of epidemics [18]. Individuals who perceive a high disease threat pay more attention to a potential disease threat and make a stronger response to it.

### 1.2. Hypothesis Development

There may exist a relationship between perceived infectability and rumor spreading. Perceived infectability refers to an individual’s beliefs about susceptibility to infectious diseases, which is used to assess perceptions of future health vulnerability and is an important indicator of perceived disease threat [19]. High perceived infectability implies a higher level of concern about a disease, as well as more anxiety and fear in the context of an epidemic [20,21]. Anxiety and fear are important pre-influencing factors of rumor spreading [7,8,22]. In addition, a disease threat affects the conformity of individuals. It has been found that, after receiving disease threat initiation, subjects show a higher tendency to subordinate attitudes and behaviors [23]. Rumor spreading, on the other hand, has obvious conformity characteristics when individuals spread unconfirmed but widely circulated information. Individuals with high perceived infectability experience a higher threat of disease in the COVID-19 outbreak and are more likely to engage in rumor spreading due to conformity. Thus, we propose hypothesis 1: Perceived infectability significantly and positively predicts rumor spreading.

Individuals with high perceived infectability are more likely to believe rumors. Rumors that spread during an epidemic convey a great deal of information to the general public about the epidemic, including how the virus is transmitted, the extent of transmission and how to prevent it [24]. In other words, these rumors often contain potential disease threats or provide ways to cope with the disease directly or indirectly. According to the smoke detector principle of the behavioral immune system, the behavioral immune system can predispose individuals to oversensitivity or overgeneralization to disease-related signals [11,15]. In the context of an epidemic, individuals with high perceived infectability tend to be more sensitive to disease information provided by rumors and are more likely to choose to believe them, ultimately leading to disease avoidance. Similarly, previous studies have found that the behavioral immune system leads to individuals’ tendency to conformity behavior [23], more conservative political attitudes [25] and more devout religious beliefs [26], and that such cognitive tendencies contribute to reducing individuals’ risk of disease infection. Therefore, we propose hypothesis 2: Perceived infectability significantly and positively predicts rumor trust.

Rumor trust may lead to more rumor spreading. Research related to the motivation of rumor spreading suggests that individuals engage in rumor spreading to enhance their self-worth or to establish and maintain good social relationships [27]. Therefore, individuals are more likely to spread rumors that they identify as true rather than rumors that they identify as false. Once people spread rumors that they are not sure about, they lose credibility in the eyes of the rumor recipient, which is detrimental to the expression of self-worth and the establishment of social relationships. Thus, people usually choose to stop spreading rumors when they are not sure about them [28]. Numerous empirical studies have also found that, if more individuals believe in rumors, they are more likely to spread those rumors [7,22,29,30,31,32]. Therefore, we propose hypothesis 3: Perceived infectability indirectly influences rumor spreading through the mediating role of rumor trust.

In summary, we attempted to verify the effect of perceived infectability on rumor spreading and the mediating role of rumor trust through two studies. Study 1 tested the hypotheses of this study through scale measurement. Considering the possible shortcomings of the scale, we took an alternative approach in Study 2 to validate the variable relationships again to improve the reliability of the results. Study 2 further tested the findings of Study 1 through the recall–report task of rumor spreading.

## 2. Study 1

### 2.1. Study 1 Methods

#### 2.1.1. Participants

The convenient sampling method was used to recruit participants through the online questionnaire platform Wenjuanxing, which is equivalent to Qualtrics and provides online questionnaire design and survey functions for companies, research institutions and individuals. A total of 661 people (254 male; *M*_age_ = 33.39), mainly from central and southern China, participated in this survey in exchange for a small payment. All participants agreed to participate in the study after reading an online consent form, and no one reported that they were suffering from COVID-19.

#### 2.1.2. Measures

##### Perceived Infectability

The perceived infectability subscale of the Perceived Vulnerability to Disease Scale developed by Duncan et al. was used [19]. It consists of seven items, such as “In general, I am very susceptible to colds, flu, and other infectious diseases”. All items were rated on a seven-point scale ranging from 1 (completely disagree) to 7 (completely agree), and some questions were reverse-scored. A higher score indicates that an individual believes that he or she is more susceptible to infectious diseases. Cronbach’s alpha for the scale was 0.70.

##### Rumor Trust

Based on the definition of rumors in previous studies [3,9], two self-created items were used: “I believed some information in the COVID-19 epidemic when it was not known to be true or false” and “I believed some information that was later proven to be false”. A 5-point Likert scale was used, with 1 = never and 5 = always. Higher scores indicate higher levels of trust in rumors among individuals. Cronbach’s alpha for the scale in this study was 0.76.

##### Rumor Spreading

The Rumor Spreading Questionnaire developed by Hu et al. was adopted [9]. It has 4 items, 2 of which were used to measure the spreading behavior of the subjects when they did not know whether the information was true or false, and two items were used to measure subjects’ spreading behavior when they believed the information was not true. All responses ranged from 1 = never to 5 = always. The scale was proved to have good reliability and validity among Chinese Internet users [10]. Cronbach’s alpha for the scale was 0.85.

#### 2.1.3. Data Analysis

First, because all data were collected with questionnaires, we used Mplus 8.3 (Muthen & Muthen, Los Angeles, CA, USA) for the common method bias test. Next, descriptive statistics and correlation analysis were performed using SPSS 25.0 (IBM, Armonk, NY, USA). Finally, Mplus 8.3 was used to test the hypothetical model, and the bootstrap method, which can estimate both direct and indirect effects, was further used to conduct mediating effect analysis. In addition, referring to previous studies [8,29,33], the current study included gender, age, education level, occupation, time spent online, and location during the epidemic as control variables in the analysis.

### 2.2. Study 1 Results and Discussion

Confirmatory factor analysis was conducted to test the hypothesis that a single factor could explain all the variance in the study data according to recommendations [34]. The results show that the model fit was poor (*χ*^2^/*df* = 13.70, *CFI* = 0.51, *TLI* = 0.42, *RMSEA* = 0.14), indicating that there was no serious problem of common method bias in this study.

The descriptive statistics and correlation analysis results are presented in Table 1. As can be seen, perceived infectability, rumor trust and rumor spreading were significantly and positively correlated with each other.

**Figure 1:**
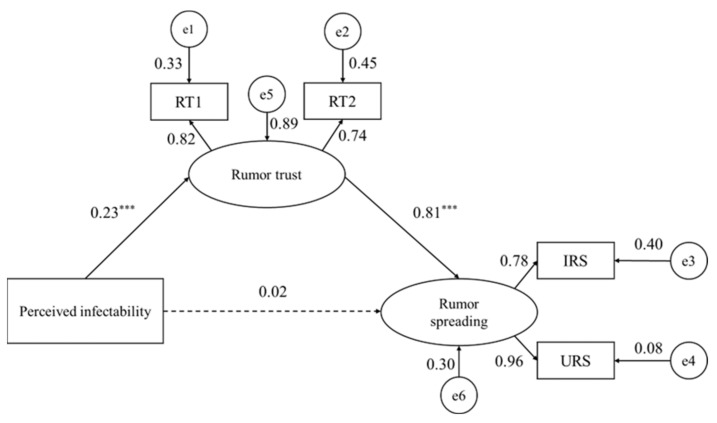
Standardized path coefficients for the mediation model. RT = Rumor trust, IRS = Intentional rumor spreading, URS = Unintentional rumor spreading. *** *p* < 0.001.

Structural equation models were constructed with Mplus 8.3 to test the mediating effect of rumor trust between perceived infectability and rumor spreading, controlling for gender, age, education level, occupation, time spent online and location during the epidemic. The results show that the model had a good fit (*χ*^2^/*df* = 1.16, *TLI* = 0.99, *CFI* = 0.99, *RMSEA* = 0.02, *SRMR* = 0.02). Perceived infectability (β = 0.23, *SE* = 0.04, *p* < 0.001) significantly predicted rumor trust. Rumor trust (β = 0.81, *SE* = 0.03, *p* < 0.001) significantly predicted rumor spreading, and perceived infectability (β = 0.02, *SE* = 0.03, *p* > 0.05) did not directly predict rumor spreading (see Figure 1). Furthermore, using the bootstrap method, the mediating effect was tested under the condition of 5000 samples, and the results show that the indirect effect of “perceived infectability → rumor trust → rumor spreading” was 0.19, accounting for 89.37% of the total effect (see Table 2).

In Study 1, frequency was used as the measurement index of rumor trust and rumor spreading to preliminarily verify our hypothesis. The results indicate that perceived infectability has a predictive effect on rumor spreading, and this effect is not direct but is mainly mediated through rumor trust. Considering that an immature self-created questionnaire was used for measurement in Study 1, to better test the stability of the results, Study 2 used the recall–report task of rumor spreading to explore the effect of perceived infectability on rumor trust and rumor spreading, and it attempted to replicate the results of Study 1 through regression analysis.

## 3. Study 2

### 3.1. Study 2 Methods

#### 3.1.1. Participants

Participants were recruited using a random sampling method through the online platform Credamo. It is akin to a Chinese version of Amazon’s Mechanical Turk Service, in which participants are paid for participating in the survey. All subjects read the consent form before the study began. A total of 10 participants were excluded because they did not pass the screening question or reported messages that were not a rumor. We obtained valid data for 180 participants (98 male; *M*_age_ = 30.46), aged 18 to 55 years old.

#### 3.1.2. Procedure

Before the formal start of the study, 8 people aged 22–55 were surveyed with the convenient sampling method to help us identify the number of rumors that participants were generally able to recall. All of them said that they could accurately recall at least three rumors in the early stage of the outbreak of COVID-19, as well as the degree of their belief in the rumors and the scale of their spread. In the formal survey, all subjects filled in the Perceived Infectability Scale first. Next, according to previous studies [28,30,35], each participant was asked to recall and report three rumors heard at the beginning of the COVID-19 outbreak. Participants were told that a rumor was defined as information that had not been officially confirmed when first heard, regardless of whether it was later proven to be true or false. Then, for each message, the participants reported how much they initially believed it and how much they spread it at the time.

#### 3.1.3. Measure

##### Perceived Infectability

This study used the same scale as that used in Study 1. In this study, Cronbach’s alpha for the scale was 0.91.

##### Rumor Trust

A question developed by Pezzo and Beckstead was used to measure rumor trust [30]. Participants were asked, “How strongly did you believe this rumor when you first heard it?” The question was scored on a 7-point Likert scale, with 1 = not at all and 7 = completely. The scores of the three rumors were summed, and a higher total score indicated a greater degree to which an individual trusted the rumor. After three reports, Cronbach’s alpha for the question was 0.72.

##### Rumor Spreading

We adapted a 7-point question developed by Wang et al. to measure rumor spreading [31]. Participants were asked, “How many people did you talk to about this rumor?” (1 = nobody, 7 = everybody I know and talk to). The scores of the three rumors were summed, and a higher total score indicated a higher degree of rumor spreading. After three reports, Cronbach’s alpha for the question was 0.80.

#### 3.1.4. Data Analysis

SPSS 25.0 was used for descriptive statistics and an independent samples *t* test. The PROCESSv3.4.1 macro program developed by Hayes was applied to test the mediation model. Consistent with Study 1, Study 2 also included gender, age, education, occupation, time spent online and location during the epidemic as control variables in the analysis.

### 3.2. Study 2 Results and Discussion

Participants with perceived infectability scores above and below one standard deviation were, respectively, selected for inclusion in the high (*n* = 39) and low (*n* = 38) perceived infectability subgroups. The results of the independent samples *t* test showed that the high perceived infectability group (*M* = 5.55, *SD* = 0.43) scored significantly higher on rumor trust and rumor spreading than did the low perceived infectability group (*M* = 2.17, *SD* = 0.25; see Table 3).

We used model 4 in SPSS macro PROCESS to test the mediating effect of rumor trust between perceived infectability and rumor spreading, controlling for gender, age, education level, occupation, time spent online and location during the epidemic. The results showed that perceived infectability significantly predicted rumor trust (β = 0.31, *SE* = 0.07, *p* < 0.001). Rumor trust significantly predicted rumor spreading (β = 0.46, *SE* = 0.07, *p* < 0.001), and perceived infectability did not directly predict rumor spreading (β = 0.07, *SE* = 0.07, *p* > 0.05). The results of the mediation effect analysis show that the indirect effect of “perceived infectability → rumor trust → rumor spreading” was 0.14, accounting for 66.01% of the total effect (0.22), with a Bootstrap 95% confidence interval of [0.07, 0.22] (see Table 4).

The results in Study 2 were consistent with those of Study 1 after adopting the recall–report task of rumor spreading. This again manifested that perceived infectability is a predictor variable of rumor spreading, but this predictive role is mainly achieved through the mediating role of rumor trust.

## 4. Discussion

In this study, with the outbreak of COVID-19 as the background, we investigated the influence of perceived infectability on rumor spreading in the context of a high-intensity disease threat. Both studies confirmed that perceived infectability significantly predicted rumor trust, which then influenced rumor spreading, and that its effect on rumor spreading was mainly mediated by rumor trust. This study is the first to identify the effect of perceived infectability on rumor spreading and to explain “how it works” from the perspective of behavioral immune system theory. The results not only advance the understanding and application of the behavioral immune system but also explore the new factor of personal traits that influence rumor spreading in public health emergencies, which is the innovation of this study.

### 4.1. Main Findings

First, this study found that perceived infectability significantly and positively predicted rumor trust. Previous studies on the behavioral immune system have found that the activation of the behavioral immune system affects people’s perceptions, such as prejudice against the elderly, obese and disabled [12,13,14], a preference for symmetrical faces [36], harsher condemnation of unethical behavior [37], a more conformist attitude [23], more conservative political attitudes [25] and more devout religious beliefs [26]. Some of these conclusions were also verified in the context of COVID-19 [16]. These cognitive changes are essentially adaptive cognitions that individuals develop in the face of disease threats to protect themselves from disease. The results of the present study also suggest that the activation of the behavioral immune system may lead to another cognitive tendency of individuals in the presence of an epidemic or even a public health emergency, namely rumor trust. When a rumor conveys information about a potential disease threat, choosing to believe it helps individuals take better measures to prevent it, whereas choosing to ignore it may pose a risk of infection. Since humans are direct carriers of disease, the smoke detector principle explains why activation of the behavioral immune system leads to biases against aging, obesity and disability. However, various unconfirmed epidemic-related messages spread on social media have quietly become indirect carriers of disease threats. The present study attempted to transfer the phenomenon of the smoke detector principle to such rumor messages, and it was confirmed.

Second, rumor trust significantly and positively predicted rumor spreading. Because humans always tend to behave in a manner consistent with their perceptions, rumors perceived as true are more likely to be spread [38]. Moreover, spreading true information is more conducive to self-worth and social bonding. Early scholars did not examine the role of rumor trust on rumor spreading, but they argued that, sometimes, obviously false rumors could be spread [39]. However, in recent years, the relationship between rumor trust and rumor spreading has been continuously confirmed by studies in different contexts [7,22,29,30,31,32], and the present study similarly validates this view in the context of public health emergencies.

As a proximal factor affecting rumor spreading, rumor trust has always been tested as a mediating variable. For example, Kim found that high virality metrics can affect rumor spreading through rumor trust [29]. Guo et al. believed that rumor trust plays a mediating role between information acquisition models and rumor spreading [32]. Sun et al. confirmed that the presence of opinionated leaders makes people trust rumors more and then spread rumors [7]. It can be found that previous studies tend to discuss the influence of the characteristics of rumors on their credibility, i.e., “What kind of rumors make people believe more”. This study, however, focuses on “what kind of people are more likely to believe rumors”. The results of this study expand our understanding of trait factors affecting rumor spreading.

### 4.2. Implications of this Study

From the perspective of rumor spreading, in the context of COVID-19, studies on influencing factors of rumor spreading and their mechanisms mainly focus on cognition and emotion, such as the role of anxiety [7], fear [8], risk perception [10], information source credibility [40], etc. Nevertheless, there are still some shortcomings in the existing studies.

First, personal traits as antecedent influencing factors of cognition and emotion have not been emphasized, and it is necessary to study what kinds of people are more likely to have cognitive biases and experience disease-related negative emotions. Second, with the COVID-19 epidemic as the background, existing studies have verified the results of previous studies on rumors based on life events, social security accidents and other situations. However, there exist new characteristics in public health emergencies, such as a wide range of disease threats and invisible virus transmission, so there may be new influencing factors that have not been considered. In this study, starting with the behavioral immune system, we identified a new factor affecting rumor spreading in the context of an epidemic, namely perceived infectability. As an important factor in the trait activation of the behavioral immune system, perceived infectability was confirmed to be a solid personal trait [18], which may further influence rumor spreading by affecting individual cognition and emotion. This not only provides ideas for this study but also provides space for further research.

In addition, the results of this study suggest that individual differences and resulting cognitive differences should be considered in the management of rumors spreading in public health emergencies. With the development of web technology, accurate algorithms can push information to groups with similar characteristics [41]. For example, people who believe they are more susceptible to disease have more information-seeking behaviors [42] and more serious panic buying behaviors [43], and studies have also found that people who are worried about contracting COVID-19 spend more money on toilet paper [44]. These behavioral data could leave a trail on the web, and big data algorithms can be used to locate these target groups. Based on these interesting findings, big data can be used to promote rumor-debunking platforms and provide rumor-debunking information to these target groups to reduce their trust in rumors, thus reducing rumor spreading behavior.

### 4.3. Limitations

There are some limitations in the present study. First, although this study adopted some measures, such as reverse scoring and pre-surveying before the formal test, both studies used methods in which participants self-reported, which could still lead to some bias. More objective data can be used to further verify the results of this study. Second, both studies used cross-sectional data, and caution is needed in interpreting causality. Third, in Study 1, we used an immature self-compiled scale, and although we hoped to compensate for this with the recall–report task in Study 2, memory is not always completely reliable. Fourth, based on theoretical derivation, although there was a wide variety of rumors during the epidemic, our findings may be more applicable to rumors of disease cues that are highly relevant to individuals. Future studies can classify types of rumors to test the applicability of the conclusions of the present study.

## 5. Conclusions

Our findings suggest that people with high perceived infectability were more likely to believe and spread rumors during the COVID-19 pandemic. Rumor trust plays an important mediating role between perceived infectability and rumor spreading.

## Figures and Tables

**Table 1:** Descriptive statistics and correlations among variables in Study 1.

Variables	*M*	*SD*	1	2	3	4	5	6	7	8
1. Gender	0.38	0.49								
2. Age	33.39	13.82	0.21 ***							
3. Education level	2.69	1.12	0.07	−0.23 ***						
4. Occupation	1.53	1.53	0.10 **	0.67 ***	−0.33 ***					
5. Time spent online	2.33	1.12	−0.05	−0.35 ***	0.36 ***	−0.31 ***				
6. Location	2.43	0.74	−0.02	−0.03	−0.01	−0.10 **	0.02			
7. Perceived infectability	3.80	0.97	−0.06	0.10 *	−0.14 **	0.06	−0.06	−0.01		
8. Rumor trust	2.48	0.85	0.01	−0.17 ***	0.12 **	−0.14 ***	0.14 ***	0.05	0.17 ***	
9. Rumor spreading	1.87	0.78	0.15 ***	−0.14 ***	0.18 ***	−0.16 ***	0.15 ***	0.03	0.16 ***	0.66 ***

Gender, age, education level, occupation, time spent online and location were treated as dummy variables; * *p* < 0.05, ** *p* < 0.01, *** *p* < 0.001.

**Table 2:** Mediating effect analysis in Study 1.

Pathways	Point Estimate	*SE*	BC Bootstrap 95% CI	
			Lower	Upper
Total effects:				
Perceived infectability → Rumor spreading	0.21	0.04	0.13	0.28
Indirect effects:				
Perceived infectability → Rumor trust → Rumor spreading	0.19	0.04	0.12	0.26
Direct effects:				
Perceived infectability → Rumor spreading	0.02	0.03	−0.05	0.09

**Table 3:** Independent samples *t* test in Study 2.

Variables	High Perceived Infectability	Low Perceived Infectability	*t*	*p*
Rumor trust	4.74 ± 1.22	3.38 ± 1.43	4.49 ***	0.000
Rumor spreading	5.08 ± 1.32	3.88 ± 1.66	3.50 **	0.001

** *p* < 0.01, *** *p* < 0.001.

**Table 4:** Regression analysis of the mediation model in Study 2.

Outcome	Predictors	*R* ^2^	*F*	β	*t*	[*LLCI, ULCI*]
Rumor trust	Perceived infectability	0.13	3.56 **	0.31 ***	4.91	[0.17, 0.46]
Rumor spreading	Perceived infectability	0.26	7.32 ***	0.07	1.04	[−0.07, 0.21]
	Rumor trust			0.46 ***	6.51	[0.32, 0.60]

** *p* < 0.01, *** *p* < 0.001.

## Data Availability

Data will be provided by the authors on request.
